# CD4^+^ T Cells Mediate the Development of Liver Fibrosis in High Fat Diet-Induced NAFLD in Humanized Mice

**DOI:** 10.3389/fimmu.2020.580968

**Published:** 2020-09-11

**Authors:** Zhisheng Her, Joel Heng Loong Tan, Yee-Siang Lim, Sue Yee Tan, Xue Ying Chan, Wilson Wei Sheng Tan, Min Liu, Kylie Su Mei Yong, Fritz Lai, Erica Ceccarello, Zhiqiang Zheng, Yong Fan, Kenneth Tou En Chang, Lei Sun, Shih Chieh Chang, Chih-Liang Chin, Guan Huei Lee, Yock Young Dan, Yun-Shen Chan, Seng Gee Lim, Jerry Kok Yen Chan, K. George Chandy, Qingfeng Chen

**Affiliations:** ^1^Institute of Molecular and Cell Biology, Agency for Science, Technology and Research (A^∗^STAR), Singapore, Singapore; ^2^Genome Institute of Singapore, Agency for Science, Technology and Research (A^∗^STAR), Singapore, Singapore; ^3^Programme in Emerging Infectious Diseases, Duke-NUS Graduate Medical School, Singapore, Singapore; ^4^Key Laboratory for Major Obstetric Diseases of Guangdong Province, The Third Affiliated Hospital of Guangzhou Medical University, Guangzhou, China; ^5^Department of Pathology and Laboratory Medicine, KK Women’s and Children’s Hospital, Singapore, Singapore; ^6^Cardiovascular and Metabolic Disorders, Duke-NUS Graduate Medical School, Singapore, Singapore; ^7^Laboratory of Molecular Physiology, Infection and Immunity Theme, Lee Kong Chian School of Medicine, Nanyang Technological University, Singapore, Singapore; ^8^Translational Biomarkers, Merck Research Laboratories, MSD, Singapore, Singapore; ^9^Division of Gastroenterology and Hepatology, National University Hospital, National University Health System, Singapore, Singapore; ^10^Department of Reproductive Medicine, KK Women’s and Children’s Hospital, Singapore, Singapore; ^11^Experimental Fetal Medicine Group, Yong Loo Lin School of Medicine, National University of Singapore, Singapore, Singapore; ^12^Department of Physiology, Yong Loo Lin School of Medicine, National University of Singapore, Singapore, Singapore

**Keywords:** humanized mouse model, NAFLD, NASH, liver fibrosis, CD4^+^ T cells

## Abstract

Non-alcoholic fatty liver disease (NAFLD) has been on a global rise. While animal models have rendered valuable insights to the pathogenesis of NAFLD, discrepancy with patient data still exists. Since non-alcoholic steatohepatitis (NASH) involves chronic inflammation, and CD4^+^ T cell infiltration of the liver is characteristic of NASH patients, we established and characterized a humanized mouse model to identify human-specific immune response(s) associated with NAFLD progression. Immunodeficient mice engrafted with human immune cells (HIL mice) were fed with high fat and high calorie (HFHC) or chow diet for 20 weeks. Liver histology and immune profile of HIL mice were analyzed and compared with patient data. HIL mice on HFHC diet developed steatosis, inflammation and fibrosis of the liver. Human CD4^+^ central and effector memory T cells increased within the liver and in the peripheral blood of our HIL mice, accompanied by marked up-regulation of pro-inflammatory cytokines (IL-17A and IFNγ). *In vivo* depletion of human CD4^+^ T cells in HIL mice reduced liver inflammation and fibrosis, but not steatosis. Our results highlight CD4^+^ memory T cell subsets as important drivers of NAFLD progression from steatosis to fibrosis and provides a humanized mouse model for pre-clinical evaluation of potential therapeutics.

## Introduction

Over the past few decades, the prevalence of non-alcoholic fatty liver disease (NAFLD) – an array of progressive clinical presentations including non-alcoholic fatty liver (NAFL), non-alcoholic steatohepatitis (NASH), liver fibrosis and cirrhosis, liver failure, and hepatocellular carcinoma (HCC) – has increased substantially, surpassing other liver conditions (e.g., viral hepatitis and alcoholic liver disease) as the leading cause of liver-related morbidity and mortality (1). With a global prevalence of ∼25%, NAFLD is positively correlated with a country’s economic status (2). While NAFLD is a major healthcare problem in Western countries, epidemiological studies indicate that NAFLD equally affects the Middle East, Far East, Africa, the Caribbean, and Latin America (3). The global surge in NAFLD is due to its strong pathophysiological association with diabetes and obesity, both of which have reached epidemic proportions. Given that there is no established therapy to curb its progression, NAFLD can potentially become a major health burden.

Fat accumulation in the liver (>5% of hepatocytes) that is independent of excessive alcohol intake is a hallmark of NAFLD (1). While NAFL is benign and reversible by adopting healthier lifestyle changes, when left uncontrolled, it can progress to NASH, a severe disease characterized by hepatocellular ballooning degeneration, inflammation and fibrosis (4). NASH is irreversible and potentially fatal as it increases the risks of cirrhosis, liver failure, and HCC (5). The pathogenesis of NAFLD is not fully elucidated but is hypothesized to occur through a “multi-hit” manner. It begins with lipid accumulation in hepatocytes. Subsequently, oxidative stress, increased release of free fatty acid from adipocytes, higher levels of pro-inflammatory cytokines (TNFα, IL-6, leptin, and resistin), and decreased adiponectin collectively drive the development of liver steatosis and inflammation (5). Disease progression beyond NAFL (i.e., NASH, fibrosis, etc.) has been shown to be promoted by the generation of hepatic reactive oxygen species (ROS), macrophage activation, transforming growth factor beta 1 (TGF-β1)-mediated collagen deposition, imbalance between Th17 and regulatory T cells (Treg), and metabolic activation of intrahepatic CD8^+^ T cells and natural killer T cells (NK-T) (6, 7).

Animal models of NAFLD – genetically engineered, dietary, chemical, or a combination of these – are widely used to elucidate the pathogenesis of NAFLD and to examine the therapeutic effect of pharmacological agents (4, 6, 8–16). In mice, NAFLD-like liver pathology can be induced by a diet rich in fats and carbohydrates (4, 9, 14, 15). Although these animal models have provided valuable insights into the development of NAFLD, recent transcriptomic analysis highlighted substantial discrepancy in liver gene expression patterns with respect to NAFLD patients (17). Furthermore, even though the diet-induced DIAMOND mouse model of NAFLD recapitulates important transcriptomic (among other biological parameters) changes of progressive NASH in humans (15), it and other small animal models do not comprehensively define the role of different (human) immune subsets in NAFLD pathogenesis (4, 6, 8–16).

Here, we use mice (NOD-*scid IL2rγ^*null*^* mice engrafted with human CD34^+^ fetal liver cells) reconstituted with a functional human immune system (HIL mice) to identify human-specific immune response(s) associated with NAFL and its progression to NASH. These humanized mice, when given *ad libitum* access to a high-calorie diet and fructose drinking water (HFHC diet) for 20 weeks, developed key pathologies (e.g., weight gain, steatosis, inflammation, and fibrosis) attributed to NAFL and NASH. NOD-*scid IL2rγ^*null*^* (NSG) mice fed with HFHC diet developed liver steatosis but not inflammation and fibrosis, highlighting the importance of lymphocytes in disease progression. Our study identified human CD4^+^ T cells as a key player in promoting liver steatosis-fibrosis progression. Longitudinal analysis of peripheral blood showed that central memory and effector memory CD4^+^ T cells expanded with time and infiltrated the liver. Furthermore, *in vivo* depletion of human CD4^+^ T cells abrogated pro-inflammatory cytokines production and fibrosis. Parallel studies on patients with NAFLD showed an increase in CD4^+^ memory T cells at sites of fibrosis within the liver. These results highlight the importance of CD4^+^ memory T cells in the progression from steatosis to fibrosis in NAFLD.

This study advances our understanding of the role of the human immune system in NAFLD pathogenesis and will aid in the identification of biomarkers and novel therapeutic targets for the disease. Our model could also potentially enable pre-clinical assessments of novel immunomodulatory therapeutics for NAFLD.

## Materials and Methods

### Human Fetal Liver Progenitor Stem Cells and Tissues

Prior to collection of human samples, written consent was obtained from patients. The study conformed with the ethical guidelines of the 1975 Declaration of Helsinki and was approved by the local ethics committee of KK Women’s and Children’s Hospital (Singapore; CIRB Ref: 2013/837/D), National University Hospital (Singapore; NHG DSRB Ref: 2014/00231), and Gleneagles Hospital (Singapore; NUS-IRB Ref: 13-273E). Fetal liver tissues were freshly isolated from aborted fetuses at 15–23 weeks of gestation and processed as described previously (18). Human CD34^+^ cells were isolated and purified using EasySep^TM^ Human CD34-Positive Selection Kit (STEMCELL Technologies, Vancouver, Canada) under sterile conditions, according to manufacturer’s instructions. The purity of the CD34^+^ cells was 90–99% as determined by flow cytometry. Healthy liver perfusates were obtained from living donor liver transplantations (Asian American Liver Centre, Gleneagles Hospital, Singapore) as described previously (19), by flushing intrahepatic veins with cold saline prior to transplantation. Liver explants from patients with advanced cirrhosis and/or HCC were analyzed (Division of Gastroenterology & Hepatology, National University Hospital, Singapore). The non-cirrhotic/HCC, fatty liver portion was cut into small pieces and incubated in RPMI medium (Sigma-Aldrich, St. Louis, MO, United States) supplemented with DNAse I (1 μg/mL; Roche, Basel, Switzerland), collagenase IV (0.8 mg/mL; Sigma-Aldrich) and 10% FBS (Thermo Fisher Scientific, Waltham, MA, United States) at 37°C for 1 h. Leukocytes were isolated from the supernatant fraction after centrifugation at 50 *g* for 5 min at 4°C.

### Mice

NOD-*scid Il2rγ^*null*^* (NSG) mice were purchased from The Jackson Laboratory (stock number 005557). All mice were bred and kept under pathogen-free conditions on controlled 12-h light-dark cycle. Female and male mice were used indiscriminately. Mouse experiments and procedures were approved by the Institutional Animal Care and Use Committee (IACUC number 181367) of A^∗^STAR in accordance with the guidelines of Agri-Food and Veterinary Authority and the National Advisory Committee for Laboratory Animal Research of Singapore.

### Generation of Humanized Mice With Human Immune System (HIL Mice)

One to three-day-old NSG pups were sub-lethally irradiated at 1 Gy and engrafted with 2 × 10^5^ human CD34^+^ fetal liver cells via intrahepatic injection. Fetal liver cells from nine different donors were used to generate the HIL mice. Mice were bled from the submandibular vein at 10 weeks post-engraftment to determine the levels of human immune reconstitution via flow cytometry. Mice with more than 10% human immune cell reconstitution (calculated based on the proportion of human CD45 relative to the sum of human and mouse CD45) in the peripheral blood were used for this study. Human chimerism analysis of HIL mice at 10 weeks post-engraftment is documented in Supplementary Table S1.

### Diet, Glucose- and Insulin- Tolerance Tests, Liver Enzymes, Cholesterol, Triglycerides and Insulin Measurement

Animals were arbitrarily assigned to normal chow diet and drinking water (chow diet) or high-fat diet (Surwit diet; 58 kcal% fat; D12331; Research Diets Inc., New Brunswick, NJ, United States) and carbohydrate-enriched drinking water (42 g/L of carbohydrates; HFHC diet) as described (4). The carbohydrate-enriched drinking water was prepared by mixing drinking water with 55% fructose (Sigma-Aldrich) and 45% sucrose (Sigma-Aldrich) by weight. Glucose- and insulin- tolerance tests were conducted in mice after a 16-h fast. Fasted mice were injected with glucose (Sigma-Aldrich) and insulin (Sigma-Aldrich) at 1 g/kg and 0.75 U/kg body weight, respectively. Blood glucose levels were measured before and at 15, 30, 60, 90, and 120 min after glucose or insulin administration using ACCU-CHEK Performa Nano blood glucose meter (Roche). Plasma alanine aminotransferase (ALT), aspartate aminotransferase (AST), γ-glutamyl transferase (GGT), cholesterol and triglycerides levels were measured using the Catalyst One Chemistry Analyzer (IDEXX, Westbrook, ME, United States) following manufacturer’s instructions. Insulin levels in plasma were measured using Insulin Mouse ELISA Kit (Thermo Fisher Scientific) according to manufacturer’s instructions.

### Histology

Liver was obtained from euthanized mice at endpoint. One liver lobe was embedded in Tissue-Tek^®^ O.C.T.^TM^ Compound (Sakura Finetek, Torrance, CA, United States) and snap-frozen while the remaining liver tissue was fixed in 10% formalin. Cryosections of 5 μm were obtained from frozen liver tissue and stained with Oil Red O (Sigma-Aldrich) using established protocol. Fixed liver tissue was embedded in paraffin wax, processed to obtain 5 μm sections, and subjected to Hematoxylin & Eosin (H&E; Sigma-Aldrich), Fast Green/Sirius Red (FG/SR; Sigma-Aldrich), or immunohistochemistry (IHC) staining following established protocols. Anti-human CD4 (ab133616; Abcam, Cambridge, United Kingdom), anti-human CD8 (ab93278; Abcam), anti-human CD68 (ab955; Abcam), and anti-alpha smooth muscle actin (ab5694; Abcam) antibodies were used for IHC. TUNEL staining was performed using the ApopTag^®^ Plus Peroxidase In Situ Apoptosis Detection kit (Merck, Kenilworth, NJ, United States) according to the manufacturer’s instructions. Histopathological images were acquired using Axio Scan. Z1 slide scanner (Zeiss, Oberkochen, Germany) and analyzed using Zen 2 (blue edition; Zeiss) software. Histological NAFLD was scored using the grading system established by Liang et al. (20). Briefly, the extents of steatosis (defined as macrovesicular steatosis and microvesicular steatosis), hypertrophy, and inflammation (defined as a cluster of more than five immune cells per focus) were examined on H&E stained liver cross-sections at 5 × magnification. Steatosis was scored 0–3 based on the percentage of area affected: 0 (<5%), 1 (5–33%), 2 (33–66%), and 3 (>66%). Inflammation was evaluated at five different image fields and was scored 0–3 based on the average number of immune foci per field: 0 (0.5 foci), 1 (0.5–1.0 foci), 2 (1.0–2.0 foci), and 3 (>2.0 foci). A summary on the distribution of histological NAFLD scores of mice is documented in Supplementary Table S2. The extent of fibrosis was examined on FG/SR stained slides at 5 × magnification and the percentage of the fibrotic area was determined using ImageJ software (version 1.5b).

### Total RNA Extraction and Gene Expression Analysis

Total RNA was extracted using RNeasy Mini Kit (QIAGEN, Hilden, Germany) according to manufacturer’s instructions. Quantification and reverse transcription of total RNA were done using NanoDrop 1000 Spectrophotometer (Thermo Fisher Scientific) and Quantitect reverse transcription kit (QIAGEN), respectively. qRT-PCR reactions (12.5 ng/μL of cDNA) were prepared with SsoFast^TM^ EvaGreen^®^ Supermix kit (Bio-Rad, Hercules, CA, United States) and performed on CFX96 Touch^TM^ Real-Time PCR Detection System (Bio-Rad) with the following cycling conditions: 95°C for 5 min, followed by 40 cycles of 95°C for 10 s and 60°C for 30 s, and ending with a melt curve analysis. Target genes were normalized to *GAPDH* and their relative expression analyzed using the 2^–Δ^ ^Δ^ ^*Ct*^ method. Briefly, ΔΔCt was calculated as ΔCt_*HFHC*_–ΔCt_*Chow*_, with ΔCt determined as Ct_*gene of interest*_–Ct*_*GAPDH*_*. The fold change for each gene between chow and HFHC was calculated as 2^–Δ^ ^Δ^ ^*Ct*^. The primer sequences of the human liver fibrosis genes analyzed are listed in Supplementary Table S3.

### Liver and Peripheral Blood Immune Cells Isolation

The isolation of immune cells from liver and peripheral blood has been described previously (18). Briefly, peripheral blood was collected from the submandibular vein of mice into EDTA tubes (Grenier Bio-One, Kremsmünster, Austria) and red blood cells (RBCs) were lysed using RBC lysis buffer (Thermo Fisher Scientific) prior to staining. For liver, tissues were mashed through a 100 μm cell strainer and cell debris was removed by centrifugation in 35% v/v Percoll solution (GE Healthcare, Chicago, IL, United States) and RPMI. RBCs were lysed and immune cells were passed through a 70 μm cell strainer prior to downstream analysis.

### Immunophenotyping of Leukocytes

Live immune cells from peripheral blood and liver were determined using LIVE/DEAD^TM^ Fixable Blue Dead Cell Stain kit (Thermo Fisher Scientific) following manufacturer’s protocol. Cell-specific marker staining was performed by incubating cells with anti-human CD45 (HI30; Biolegend, San Diego, CA, United States and BD Biosciences, San Jose, CA, United States), anti-human CD3 (UCHT1; Biolegend), anti-human CD4 (SK3; BD Biosciences), anti-human CD8 (SK1; Biolegend), anti-human CD45RA (HI100; Biolegend), anti-human CCR7 (3D12; BD Biosciences), anti-human HLADR (L243; BD Biosciences), anti-mouse CD45.1 (A20; Biolegend), anti-human CD14 (M5E2; BD Biosciences), anti-human CD16 (3G8; BD Biosciences), anti-human CD19 (HIB19; Biolegend), anti-human CD123 (6H6; Biolegend), anti-human CD11c (B-ly6; BD Biosciences), and anti-human CD56 (HCD56; Biolegend) for 30 min at room temperature, in two separate panels. After staining, cells were washed and resuspended in FACS buffer containing PBS, 0.2% bovine serum albumin (GE Healthcare) and 0.05% sodium azide (Merck) for flow cytometry data acquisition using a LSR II flow cytometer (BD Biosciences) running the FACSDiva software. Data was analyzed using FlowJo software (version 10; Tree Star Inc, Ashland, OR, United States). The following leukocytes were gated accordingly: CD4^+^ T cells (CD45^+^/CD3^+^/CD4^+^), CD8^+^ T cells (CD45^+^/CD3^+^/CD8^+^), naïve T cells (CD45^+^/CD3^+^/ CD4^+^ or CD8^+^/CCR7^+^/CD45RA^+^), central memory (CM) T cells (CD45^+^/CD3^+^/CD4^+^ or CD8^+^/CCR7^+^/CD45RA^–^), effector memory (EM) T cells (CD45^+^/CD3^+^/CD4^+^ or CD8^+^/ CCR7^–^/CD45RA^–^), effector memory re-expressing CD45RA (TEMRA) T cells (CD45^+^/CD3^+^/CD4^+^ or CD8^+^/CCR7^–^/ CD45RA^+^), CD14^+^ monocytes (CD45^+^/CD16^–^/CD14^+^), CD14^+^CD16^+^ monocytes (CD45^+^/CD16^+^/CD14^+^), CD16^+^ monocytes (CD45^+^/CD14^–^/CD16^+^/CD56^–^), NK cells (CD45^+^/ CD14^–^/CD56^+^), B cells (CD45^+^/CD16^–^/CD14^–^/CD56^–^/ CD19^+^), pDC (CD45^+^/CD16^–^/CD14^–^/CD56^–^/CD19^–^/ CD123^+^/CD11c^–^), and mDCs (CD45^+^/CD16^–^/CD14^–^/CD 56^–^/CD19^–^/CD123^–^/CD11c^+^).

### *In vivo* Depletion of Specific Immune Cell Subsets in HIL Mice

T cells (CD4^+^ and CD8^+^) and CD14^+^ monocytes were depleted from 10 week-old HIL mice by intravenous injection of anti-human CD4 (RPA-T4; Biolegend), anti-human CD8 (RPA-T8; Biolegend), or anti-human CD14 (M5E2; Biolegend) antibodies at an initial dose of 50 μg per mouse. Non-depleted control mice were injected with PBS. Prior to diet treatment, depletion efficiency of specific immune cell subsets was verified in peripheral blood using flow cytometry 1 week after initial depletion. Thereafter, depletion of specific immune cell subsets was maintained by weekly intraperitoneal injection of the respective depleting antibodies at a dose of 20 μg per mouse.

### Cytokine and Chemokine Quantification

Plasma cytokine levels were measured using a premixed LEGENDplex^TM^ Human Inflammation Panel (13-plex, Biolegend). The 13 cytokines and chemokines assayed simultaneously include IL-1β, IFNα, IFNγ, TNFα, MCP-1 (CCL2), IL-6, IL-8 (CXCL8), IL-10, IL-12p70, IL-17A, IL-18, IL-23, and IL-33. Preparation of samples, reagents, and immunoassay procedures were performed according to manufacturer’s instructions. Data was acquired on a LSR II flow cytometer (BD Biosciences) running the FACSDiva software and analyzed with the LEGENDplex^TM^ Data Analysis software (Biolegend) using on a five-parameter logistic curve to derive the standard curve. Levels of IL-1β, IFNα, TNFα, IL-12p70, IL-23, and IL-33 were below detection limit and excluded from subsequent analysis.

### Statistical Analysis

Statistical analysis was performed using GraphPad Prism 8.0 software (GraphPad Software, San Diego, CA, United States). Pairwise comparison was performed using two-tailed Mann–Whitney *U* test. *P* value less than 0.05 is considered statistically significant. All data are represented as mean ± standard error of mean (SEM).

## Results

### Key NAFLD-Associated Pathologies Were Recapitulated in HIL Mice Fed With HFHC Diet

**FIGURE 1:**
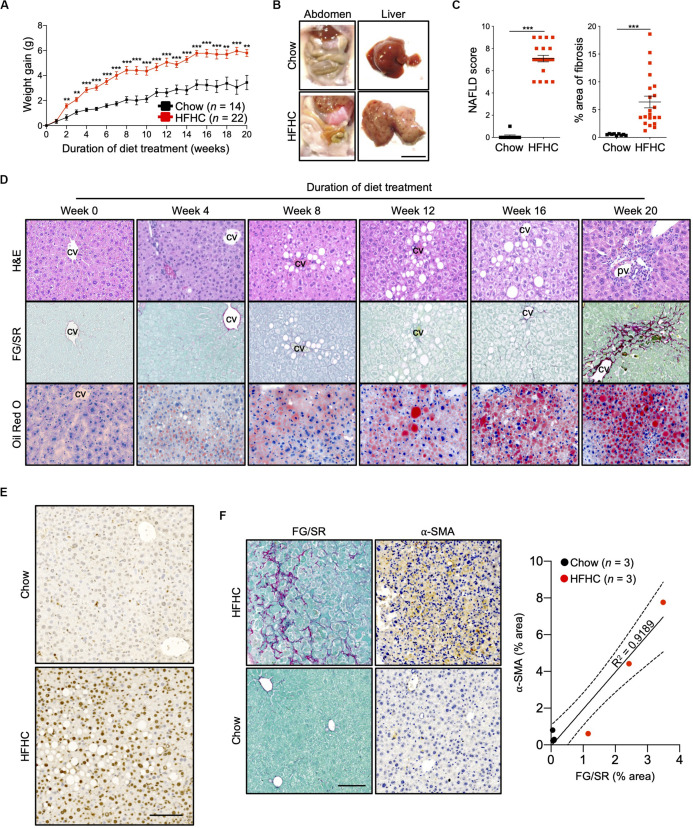
Key NAFLD associated pathologies were recapitulated in HIL mice fed with HFHC diet. Ten to 12-week-old HIL mice were given *ad libitum* access to HFHC diet over a period of 20 weeks. **(A)** Weight gain of HIL mice fed with chow diet and HFHC diet over 20 weeks. Weight gain was determined after normalizing to weight at week 0. Data are presented as mean weight gain ± SEM. Two-tailed Mann–Whitney *U* test; **p < 0.01, ***p < 0.001. **(B)** Accumulation of abdominal fat and evidence of severe liver damage in HIL mice fed with HFHC diet. Images are representative of each diet group at week 20. Scale bar: 1 cm. **(C)** Comparison at week 20 of NAFLD score and % area of liver fibrosis in HIL mice fed with chow (*n* = 9) or HFHC diet (*n* = 21). Data are presented as mean ± SEM. Two-tailed Mann–Whitney *U* test; ***p < 0.001. **(D)** H&E, Fast Green/Sirius Red (FG/SR) and Oil Red O staining of liver sections from HFHC-fed HIL mice at weeks 0, 4, 8, 12, 16, and 20 of diet treatment. Images are representative of 3–6 HIL mice per time point. Scale bar: 100 μm. cv, central vein; pv, portal vein. **(E)** TUNEL staining of liver sections from chow-fed and HFHC-fed HIL mice at 20 weeks. Scale bar: 100 μm. **(F)** Positive correlation between presence of activated hepatic stellate cells (marked by α-SMA) and proportion of fibrotic region (FG/SR staining) in liver of HIL mice at 20 weeks. Scale bar: 100 μm.

Ten to 12-week-old HIL mice were randomly assigned to two groups. The first group received a normal chow diet with drinking water (chow diet) while the second group received a high fat diet (Surwit diet; 58 kcal% fat that is mainly saturated) with carbohydrate-enriched drinking water (HFHC diet). The mice were monitored for 20 weeks. HIL mice fed with HFHC diet exhibited significant weight gain compared to HIL mice fed with chow diet (Figure 1A). Weight gain began as early as 2 weeks and continued through the 20-week study. After 20 weeks on the HFHC diet, we observed increased accumulation of abdominal fat as well as enlargement, pale coloration, and scarring of the liver (Figure 1B). Analysis of liver sections revealed centrilobular (zone 3) macrovesicular and microvesicular steatosis, periportal and/or lobular infiltration of immune cells, and perisinusoidal and periportal fibrosis corresponding to stage 2 fibrosis in human NASH (21), collectively leading to an overall higher NAFLD score in HFHC-fed compared to chow-fed HIL mice (Figures 1C,D). Notwithstanding the recapitulation of key NAFLD-associated pathologies, the frequency of hepatocellular hypertrophy and ballooning was low (Figures 1C,D and Supplementary Table S2) although there was an increased level of TUNEL-positive hepatocytes in HFHC-fed HIL mice, indicating DNA damage due to steatosis (Figure 1E). We also found activated hepatic stellate cells (marked by α-SMA) in fibrotic areas suggesting their involvement in liver fibrosis (Figure 1F).

**FIGURE 2:**
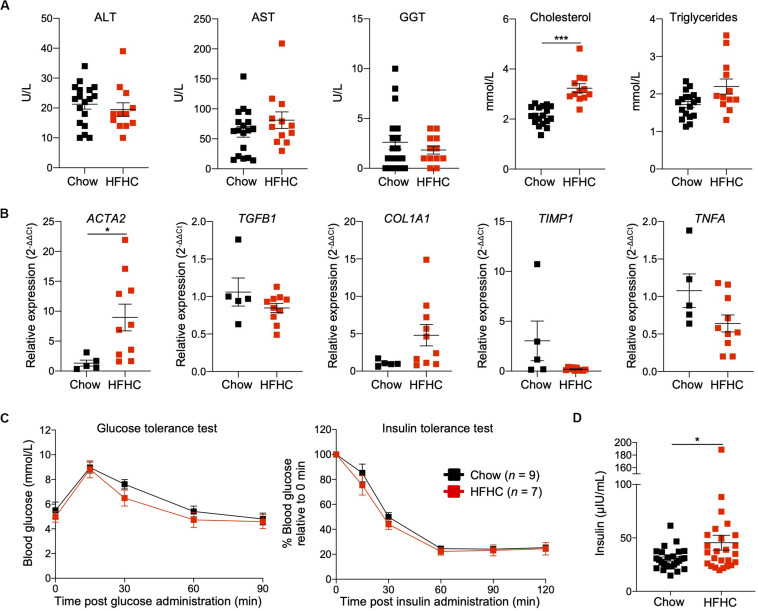
Metabolic and transcriptomic characterization of HIL mice fed with HFHC diet. **(A)** Plasma ALT, AST, GGT, and cholesterol and triglycerides at 20 weeks (Chow *n* = 18; HFHC *n* = 12). **(B)** Relative expression levels of human liver fibrosis genes *ACTA2*, *TGFB1*, *COL1A1*, and *TIMP1*, and *TNFA* in total liver RNAs from HIL mice at 20 weeks (Chow *n* = 5; HFHC *n* = 10). Gene expression is expressed as fold change compared to chow-fed mice after normalizing to *GAPDH*. **(C)** Blood glucose levels at specified time during glucose- and insulin-tolerance tests. **(D)** Insulin levels in plasma (Chow *n* = 26; HFHC *n* = 25) at 20 weeks. Data are presented as mean ± SEM. Two-tailed Mann–Whitney *U* test; *p < 0.05, ***p < 0.001.

To characterize NAFLD progression, HIL mice fed with HFHC diet were sacrificed monthly over the duration of the study. Gross morphology revealed progressive liver discoloration and scarring accompanied by abdominal fat accumulation with time (Supplementary Figure S1A). Histologically, we computed higher NAFLD scores over time due to the progressive increase of steatosis, immune cell infiltration and fibrotic areas (Figure 1D, Supplementary Figure S1B, and Supplementary Table S2). Consistent with hepatic steatosis, plasma cholesterol and triglycerides levels were moderately increased (Figure 2A). Analysis of liver fibrotic gene expression revealed an up-regulation of human alpha-smooth muscle actin (*ACTA2*), which corroborates our IHC staining of α-SMA (Figure 1F), and alpha-1 type I collagen (*COL1A1*) in HFHC-fed mice, congruous with increased liver fibrosis, although transforming growth factor beta 1 (*TGFB1*), and tissue metallopeptidase inhibitor 1 (*TIMP1*) were unchanged (Figure 2B). In addition, tumor necrosis factor (*TNFA*), an inflammation marker, was slightly down-regulated in spite of evident liver inflammation based on histological assessment (Figure 2B). Despite obvious liver damage at endpoint, there was no significant difference in plasma levels of liver enzymes γ-glutamyl transferase (GGT), alanine aminotransferase (ALT), and aspartate aminotransferase (AST) between chow- and HFHC-fed HIL mice (Figure 2A). Since NAFLD is often associated with insulin resistance, intraperitoneal glucose-, and insulin-tolerance tests were conducted. Blood glucose levels were not significantly different between chow- and HFHC-fed mice, indicating that the HFHC diet did not promote insulin resistance in HIL mice (Figure 2C). The insulin sensitivity in our model could be an adaptation to insulin resistance by increasing insulin secretion (22). Indeed, plasma insulin levels of HFHC-fed mice were significantly higher compared to their chow-fed counterparts (Figure 2D). Taken together, our results demonstrate that HIL mice on the HFHC diet develop key NAFLD-associated liver pathologies (steatosis, inflammation, and fibrosis).

### The Human Immune System Is Essential for the Development of HFHC-Induced Inflammation and Liver Fibrosis

To determine the contribution of human immune system to NAFLD development in HIL mice, we fed NSG mice (without human immune cell-engraftment) with chow or HFHC diet (Supplementary Figure S2). Like HIL mice, NSG mice on the HFHC diet experienced significant weight gain over time (Figure 1A and Supplementary Figure S2A). In contrast to the livers of HIL mice fed with HFHC diet (Figures 1B,D), livers of NSG mice maintained their red coloration with no visible scarring (Supplementary Figure S2B). Analysis of liver sections from NSG mice on the HFHC diet revealed centrilobular macrovesicular and microvesicular steatosis without inflammation or fibrosis (Supplementary Figures S2C,D and Supplementary Table S2). The difference in phenotype between HIL and NSG mice suggests that specific human immune cells are necessary to drive the progression from steatosis to liver inflammation and fibrosis. Since mature B, T, and Natural killer (NK) cells are absent in NSG mice but present in HIL mice, they are potential culprits of this immunopathological process.

### Central and Effector Memory CD4^+^ T Cells Are Expanded in Peripheral Blood of HIL Mice Fed With HFHC Diet

**FIGURE 3:**
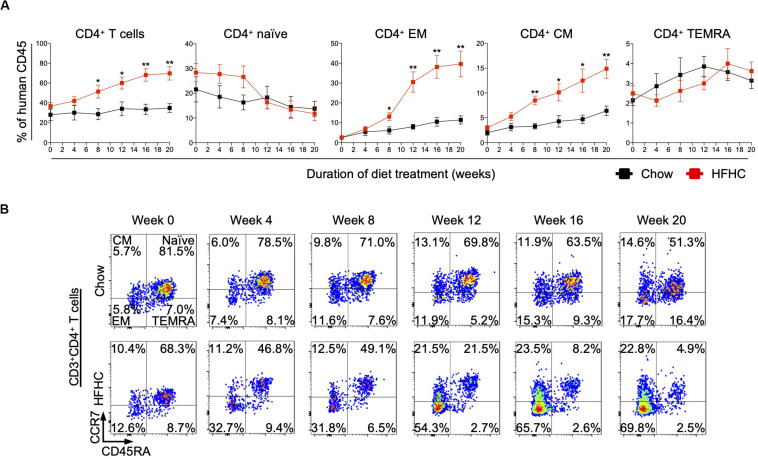
Peripheral central memory and effector memory CD4^+^ T cells increased in HIL mice fed with HFHC diet. Blood was drawn from HIL mice fed with chow diet (*n* = 7) or HFHC diet (*n* = 8) at weeks 0, 4, 8, 12, 16, and 20, and immunophenotyped using flow cytometry. **(A)** Longitudinal change in proportion of CD4^+^ T cell subsets. Data are presented as mean % relative to human CD45 ± SEM at each time point. Two-tailed Mann–Whitney *U* test; *p < 0.05, **p < 0.01. **(B)** Representative flow cytometry plots to illustrate the longitudinal change in CD4^+^ T cell naïve and memory subsets. The proportion of cells is presented as % relative to total CD3^+^CD4^+^ T cells. Human CD3^+^CD4^+^ T cells gated from human CD45^+^ cells were further gated based on CCR7 and CD45RA expression for CCR7^+^CD45RA^+^ naïve, CCR7^+^CD45RA^–^ central memory (CM), CCR7^–^CD45RA^–^ effector memory (EM) and CCR7^–^CD45RA^+^ effector memory re-expressing CD45RA (TEMRA) cells.

To identify human immune subsets that drive NAFLD progression, we used flow cytometry to examine longitudinal changes in the peripheral immune cell profile of HIL mice on the two diets (Figure 3 and Supplementary Figures S3, S4). With the exception of B cells, the change in absolute number of immune cells was not statistically significant over time for all immune subsets profiled, although CD4^+^ T cells did present a modest increase after 8 weeks (Supplementary Figures S3, S4). Nevertheless, the percentage of peripheral CD4^+^ T cells (with respect to human CD45) increased significantly in HIL mice fed with HFHC diet (Figure 3A). Within the CD4^+^ pool, central memory (CCR7^+^CD45RA^–^) and effector memory (CCR7^–^CD45RA^–^) T cells increased significantly, while naïve (CCR7^+^CD45RA^+^) T cells decreased with time (Figures 3A,B). Importantly, similar changes in peripheral CD4^+^ T cell subsets were reported previously in a cohort of 20 NASH patients (23), lending support to the validity of our humanized model. These results identify CD4^+^ T cells as possible contributors to NAFLD pathogenesis.

### HFHC Diet Induced Massive Hepatic Infiltration of Human Leukocytes in HIL Mice

**FIGURE 4:**
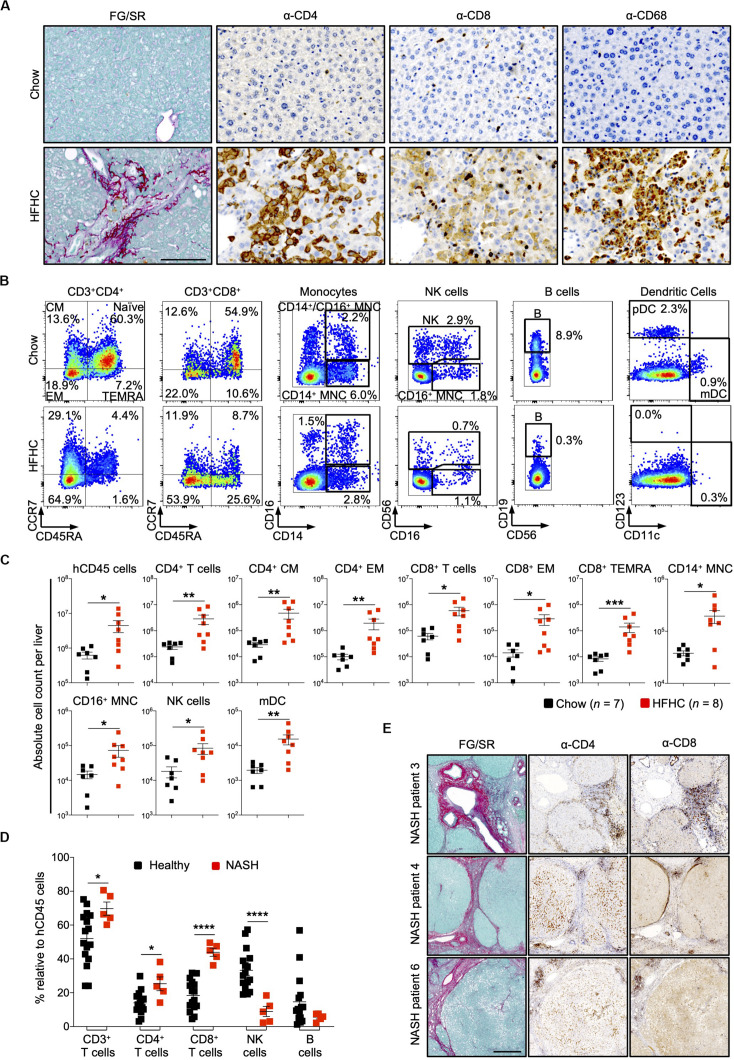
Increased intrahepatic infiltration of human leukocytes in HIL mice fed with HFHC diet. **(A)** Increased intrahepatic infiltration of CD4^+^ T cells into fibrotic region. Histological assessment of CD4, CD8, or CD68 in liver sections obtained from HIL mice fed with chow or HFHC diet. Images are representative of 5 HIL mice per diet group at week 20 from 2 independent experiments. Scale bar: 100 μm. **(B)** Representative flow cytometry plots to illustrate the change in immune profile of intrahepatic cellular infiltrates at week 20 (HFHC, *n* = 8; chow, *n* = 7). The proportion of naïve and memory T cell subsets is presented as % relative to total CD3^+^CD4^+^ or CD3^+^CD8^+^ T cells. Human monocytes (MNC: CD14^+^, CD16^+^, and CD14^+^CD16^+^), NK (CD14^–^CD56^+^), B cells (CD19^+^CD14^–^CD16^–^CD56^–^). From the CD14/CD16/CD56/CD19 negative cells, dendritic cells, pDC (CD123^+^CD11c^–^) and mDC (CD123^–^CD11c^+^) were subsequently gated. Cell proportion is presented as % relative to total human CD45 cells. **(C)** Absolute count of intrahepatic cellular infiltrates (statistically significant ones) at week 20. Data are presented as mean absolute cell count per liver ± SEM. Two-tailed Mann–Whitney *U* test; *p < 0.05, **p < 0.01, and ***p < 0.001. **(D)** Comparison of intrahepatic immune profile in liver perfusate from healthy individuals (*n* = 16) and patients with NASH (*n* = 5). Data are presented as % relative to human CD45 cells ± SEM. Two-tailed Mann–Whitney *U* test; *p < 0.05, ****p < 0.0001. **(E)** Histological assessment of CD4 and CD8 in NASH patient livers. Scale bar: 500 μm.

In HIL mice fed with chow diet for 20 weeks, human leukocytes were sparsely seen in the liver. In contrast, liver sections of HIL mice fed with HFHC diet showed a remarkable increase in human CD4^+^ and CD8^+^ T cells, and macrophages (CD68^+^), which were largely confined to fibrotic regions (Figure 4A). FACS immune profiling revealed that CD4^+^ central memory and effector memory, as well as CD8^+^ effector memory and T_*EMRA*_ (CCR7^–^CD45RA^+^) constituted majority of the T cell infiltrates within the liver (Figures 4B,C and Supplementary Figure S5A). The immune profiles of CD4^+^ T cell subsets in liver infiltrates and peripheral blood of HFHC-fed HIL mice were very similar (Figures 3, 4).

To assess the clinical relevance of our findings, we examined patients with NAFLD and compared them with healthy controls (Supplementary Table S4). Liver tissue was obtained from five patients with NAFLD (with clinical and histological evidence of NASH, cirrhosis and HCC). Perfusate of these tissues showed increased proportion of intrahepatic CD4^+^ T and CD8^+^ T cells, and decreased NK cells when compared to liver perfusate obtained from healthy donors during liver transplantation (Figure 4D). Immunohistology of liver biopsies from three NASH patients revealed accumulation of CD4^+^ and CD8^+^ T cells at fibrotic regions (Figure 4E). Collectively, these mouse and human studies suggest a role for CD4^+^ memory T cell subsets in diet-induced liver inflammation and steatosis-to-fibrosis progression. The contribution of monocytes (CD14^+^ and CD16^+^), NK cells, and myeloid dendritic cells (mDC), which were significantly elevated in HFHC-fed mice (Figure 4C), should be explored further.

### Pro-inflammatory Cytokines Were Elevated in HIL Mice Fed With HFHC Diet

**FIGURE 5:**
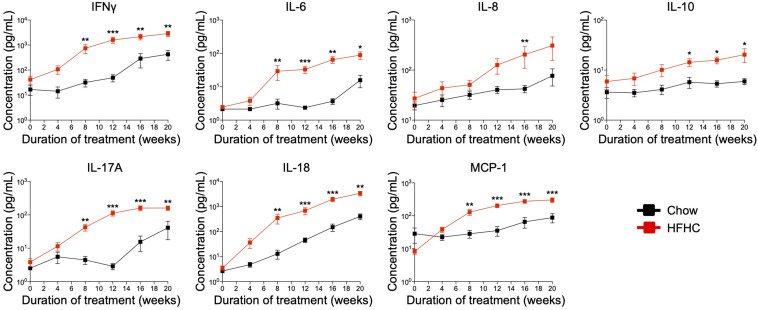
In HIL mice HFHC diet is associated with increased blood levels of inflammatory cytokines. Blood was collected monthly from HIL mice that were given *ad libitum* access to either the chow (*n* = 9) or HFHC diet (*n* = 15) over a period of 20 weeks. Plasma cytokine levels were determined using a multiplex microbead assay according to the manufacturer’s instructions. Data are presented as mean concentration ± SEM. Two-tailed Mann–Whitney *U* test; *p < 0.05, **p < 0.01, and ***p < 0.001.

Cytokine imbalance caused by excessive production of pro-inflammatory cytokines relative to anti-inflammatory cytokines is implicated in NAFLD pathogenesis (8). To identify the immune mediators that may be responsible for the pathologies induced by the HFHC diet, we examined the levels of 13 cytokines in the plasma of HIL mice fed with chow or HFHC diet (Figure 5). Six (IL-1β, IFNα, TNFα, IL-12p70, IL-23, and IL-33) of 13 cytokines were below the detection limit and therefore excluded from subsequent analysis. Plasma levels of the remaining seven were studied. These include T helper-associated/pro-inflammatory cytokines (IFNγ, IL-6, IL-17A, and IL-18), chemokines (IL-8 and MCP-1), and an anti-inflammatory (IL-10) cytokine. Before the 8th week, there was no significant difference in plasma levels of cytokines between HIL mice fed with HFHC or chow diet (Figure 5). From 8 weeks onward, cytokine levels of HFHC-fed mice were significantly higher, and this difference persisted until the 20th week (Figure 5). The increase in cytokine levels correlated with the severity of NAFLD-associated histopathology (Figure 1). The largest change was in IL-17A (∼17 fold difference), followed by IFNγ (∼11 fold), IL-18 (∼7.4 fold), IL-6 (∼4.5 fold), MCP-1 (∼4.4 fold), IL-8 (∼2.6 fold), and IL-10 (∼1.63 fold). These results highlight IL-17A and IFNγ as potentially important mediators of inflammation in NAFLD pathogenesis.

### *In vivo* Depletion of CD4^+^ T Cells Abrogated HFHC Diet-Induced Inflammatory Response and Liver Fibrosis Development

**FIGURE 6:**
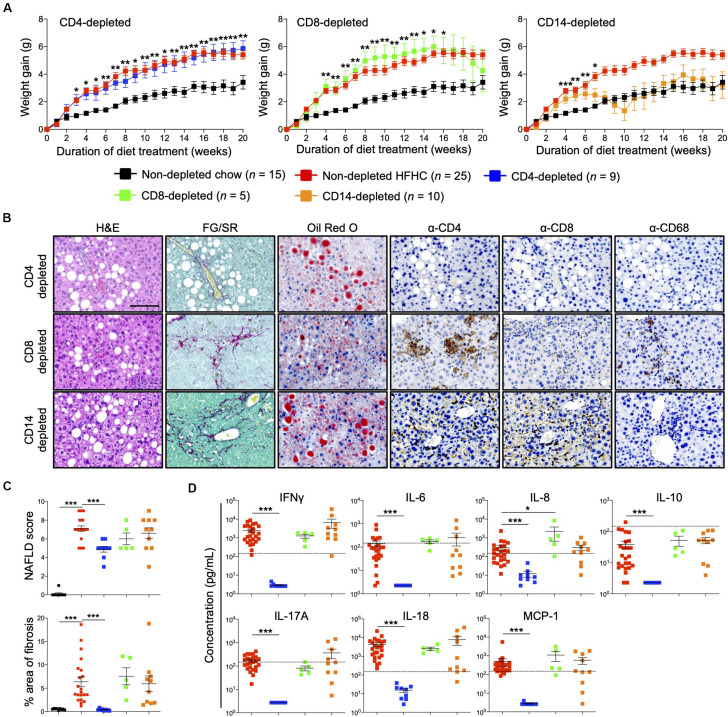
Development of HFHC diet-induced inflammation and liver fibrosis is dependent on CD4^+^ T cells in HIL mice. Ten to 12-week-old HIL mice were intravenously injected with 50 μg of anti-human CD4, CD8, or CD14 depleting antibody 1 week before start of diet treatment. Subsequently, mice were intraperitoneally injected with 20 μg of the respective depleting antibody weekly. **(A)** Weight gain of HIL mice on chow and HFHC diet (with respective immune cell depletion) over a period of 20 weeks. Weight gain was normalized to weight at week 0 and presented as mean weight gain ± SEM. Pairwise comparison between non-depleted chow and respective immune-depleted group on HFHC diet was performed using Mann–Whitney *U* test (two tail); *p < 0.05, **p < 0.01, ***p < 0.001. **(B)** Effect of immune cell depletion on HFHC diet-associated liver histopathologies. Livers of HIL mice on HFHC diet were sectioned and, respectively, stained at 20 weeks. Images are representative of 4–9 HIL mice. Scale bar: 100 μm. **(C)** Comparison of NAFLD score and % area of liver fibrosis in HIL mice fed with chow (black; *n* = 9) or HFHC diet (non-depleted HFHC; red; *n* = 21, CD4-depleted; blue; *n* = 9, CD8-depleted; green; *n* = 5, and CD14-depleted; orange; *n* = 10) at week 20. Data are presented as mean ± SEM. Pairwise comparison to non-depleted HFHC was performed using two-tailed Mann–Whitney *U* test; ***p < 0.001. **(D)** Plasma levels of inflammatory cytokines were significantly reduced in CD4-depleted HIL mice fed with HFHC diet (refer to **C** for *n*). Data are presented as mean concentration ± SEM. Pairwise comparison to non-depleted HFHC was performed using Mann–Whitney *U* test (two tail); *p < 0.05, ***p < 0.001. Dotted line represents median cytokine concentration of 15 non-depleted HIL mice fed with chow diet.

Immunophenotyping of both peripheral blood immune subsets (Figure 3) and intrahepatic cellular infiltrates (Figure 4), and evaluation of plasma cytokine levels (Figure 5), suggest that CD4^+^ T cells contribute to the progression of HFHC diet-induced NAFLD. To test this possibility, HIL mice were depleted of human CD4^+^ T cells, CD8^+^ T cells or CD14^+^ monocytes and then placed on the HFHC diet (Figure 6 and Supplementary Figure S6). Depletion of specific subsets was confirmed in peripheral blood before commencement of the dietary regimen and at 20 weeks of diet treatment (Supplementary Figure S6A). HIL mice depleted of CD4^+^ T cells (both in peripheral blood and liver) gained weight and accumulated abdominal fat like non-depleted HIL mice on the HFHC diet, which was significantly greater than non-depleted HIL mice fed with chow diet (Figure 6A and Supplementary Figure S6B). CD4^+^ T cell-depletion did not prevent hepatic steatosis, but decreased liver immune infiltration and liver fibrosis (Figures 6B,C and Supplementary Table S2). It also resulted in the depletion of intrahepatic CD8^+^ T cells and CD68^+^ macrophages, which suggests that intrahepatic infiltration of CD8^+^ T cells and CD68^+^ macrophages is dependent on CD4^+^ T cells (Figure 6B). Consequently, CD4^+^ T cell-depleted HFHC-fed HIL mice had significantly lower levels of cytokines (IFNγ, IL-6, IL-8, IL-10, IL-17A, IL-18, and MCP-1) than non-depleted HFHC-fed HIL mice (Figure 6D). Depletion of CD8^+^ T cells or monocytes did not reduce abdominal fat accumulation, hepatic steatosis, intrahepatic leukocyte infiltration, production of inflammatory cytokines, and liver fibrosis (Figures 6B–D, Supplementary Figure S6, and Supplementary Table S2). Taken together, these results demonstrate that CD4^+^ T cells, but not CD8^+^ T cells or CD14^+^ monocytes, contribute to the development of NAFLD-induced inflammation and steatosis-to-fibrosis progression.

## Discussion

Considering the pandemic scale of diabetes and obesity (risk factors for NAFLD), NAFLD poses significant health and economic burdens. A better understanding of the pathogenic mechanisms underlying disease development and progression could pave the way for new therapeutic interventions. Studies of human biological functions are typically done *ex vivo* or through clinical trials, which are limited physiologically or costly with ethical constraints, respectively (24). As such, *in vivo* studies are largely confined to animal models that may not precisely recapitulate the immunopathological changes in human diseases (25). Appropriately humanized mice could potentially circumvent these limitations by providing a clinically relevant model for assessing immunomodulatory therapeutics for NAFLD. For instance, the use of mice expressing human cytochrome P450s for pre-clinical drug testing has mitigated inter-species differences (between humans and mice) in drug metabolism and toxicological risk, and afforded better prediction of drug efficacy in human trials (26).

Here, we used NSG mice engrafted with a functional human immune system (HIL mice) to study the role of specific human immune subsets in diet induced NAFLD and its progression to NASH. HIL mice on HFHC diet not only exhibited steatosis but also features of human NASH (inflammation and fibrosis), which were importantly absent in NSG mice on the same diet. However, hepatocyte ballooning, a characteristic of human NASH, was rarely identified in our model. C57BL/6 mice fed a fast food (FF) diet exhibit pronounced hepatocyte ballooning (9). Since FF and HFHC diets both contain saturated fats and fructose, the cholesterol in the FF diet (not present in the HFHC diet) may promote hepatocyte ballooning (9). Furthermore, the grading of ballooning has been significantly subjective and debatable even amongst experts, and was hence excluded in some NAFLD scoring systems (20). Despite FF-fed C57BL/6 mice displaying high fidelity to human NASH, they still lack human immune cells (a gap that our HIL mice can fill), which are essential in the human condition. Importantly, our HFHC-fed HIL mice exhibited increased DNA damage in hepatocytes (due to lipotoxicity) that could induce liver inflammation.

Having validated the importance of the immune system in NAFLD progression, we found a significant increase in peripheral and hepatic-infiltrated human CD4^+^ T cells (central memory and effector memory) and their associated pro-inflammatory cytokines (IL-17A and IFNγ), making CD4^+^ T cells important drivers of inflammation. Activation of CD4^+^ T cells could promote infiltration of other detected immune subsets (e.g., CD8^+^ T cells and macrophages), while depletion of CD4^+^ T cells (but not CD8^+^ T cells and monocytes) abrogated immune infiltration, inflammation and fibrosis, which not only confirms our hypothesis but is consistent with previous publications about the role of CD4^+^ T cells in NAFLD progression (11, 27, 28). In a recent paper investigating the transcriptional and immune profile of NASH patients, the authors reported a NASH hepatic gene signature that showed highest enrichment of IFNγ response pathway genes (29), which concurs with the increased plasma IFNγ concentration we see in HFHC-fed HIL mice. They also similarly showed a positive correlation between subsets of blood CD4^+^ T cells (Th1 and Th17) and histological liver parameters such as lobular inflammation, ballooning and NAFLD Activity Score in NASH patients without type 2 diabetes (29). Nonetheless, the authors concluded that NASH in patients is associated with elevated intrahepatic CD8^+^ T cells (29), an observation we also made in our patient cohort. However, our mouse model suggests that intrahepatic infiltration of CD8^+^ T cells is driven by CD4^+^ T cells since depletion of the latter reduced the former and not the other way around.

Among the cytokines tested, IL-17A had the greatest fold-increase in the peripheral blood of HIL mice on the HFHC diet and decreased significantly when these mice were depleted of CD4^+^ T cells, implying that IL-17A-producing Th17 CD4^+^ T cells may contribute to steatosis-to-fibrosis progression. IL-17 axis activation and Th17/Treg cellular imbalance were previously reported to play a central role in the progression from NAFL to NASH in both mice and patients with NAFLD (11, 13, 30). An *in vitro* study showed that IL-17A enhances the expression of pro-fibrotic genes (e.g., *ACTA2* and *COL1A1*) by hepatic stellate cells through JNK-dependent up-regulation of TGF-β receptor on these cells (31), providing a possible mechanism for liver fibrosis. Furthermore, mice with defective IL-17A signaling (by IL-17A knockout or IL-17A neutralization, or by enhancing the dominance of immunosuppressive Treg) were protected from liver fibrosis and NAFLD progression (11, 32, 33). IFNγ was also increased in HIL mice on HFHC diet, which indicates that IFNγ-producing Th1 CD4^+^ T cells contribute to NAFLD progression, likely through the promotion of liver inflammation (34). Indeed, NASH patients are reported to have increased IFNγ-producing Th1 and IL-17A-producing Th17 CD4^+^ T cell subsets in peripheral blood (23, 30).

Noteworthy, a previous report showed that intrahepatic CD4^+^ T cells were reduced during methionine choline-deficient (MCD) diet-induced NAFLD in inducible liver-specific MYC oncogene (both ON and OFF) transgenic mice, although these T cells (in MYC-ON mice) were shown to be activated (CD69^+^) and to express IL-17A and IFNγ (35). *In vitro* studies on CD4^+^ T cells from MYC-ON MCD mice showed fatty acid-induced and mitochondrial ROS-mediated CD4^+^ T cell death, which reportedly led to decreased intrahepatic CD4^+^ T cells (35). While the activation of CD4^+^ T cells in Ma et al.’s model supports our findings and those of other groups who have identified the contribution of Th17 and Th1 CD4^+^ T cells in NASH inflammation, lower levels of intrahepatic CD4^+^ T cells in Ma et al.’s model compared to other NAFLD mouse models (including our HIL mouse model) may be explained by the presence of MYC oncogene in the former (11, 13, 30, 35). Ma et al. also examined archived surgical liver biopsies from patients with NASH for CD4^+^ T cells. All 16 of the patients they studied exhibited significant liver fibrosis and/or cirrhosis, indicating late stage disease. The proportion of intrahepatic CD4^+^ T cells/mm^2^ in these biopsies were similar to those in healthy controls and patients with alcoholic hepatitis, but significantly lower than in HBV/HCV-induced viral hepatitis where virus-specific infiltration of the liver is a hallmark of disease (35). In contrast, CD4^+^ T cells were increased and clustered at sites of fibrosis in the livers of the NAFLD patients (who were in the late stage of disease with evidence of NASH, cirrhosis and HCC) we studied. The difference in CD4^+^ T cell quantification may reflect the type of analysis employed. Our result was based on FACS analysis on liver perfusate while Ma et al. performed IHC and cell count on stained slides, which is dependent on the chosen image field.

Additional notable cytokines that were up-regulated in our NAFLD-HIL mouse model include IL-6, IL-8, IL-10, IL-18, and MCP-1. Up-regulation of several of these immune mediators (IL-6, IL-8, and MCP-1) was previously reported in liver cells and/or plasma of patients with steatosis and NASH, and correlated positively with the severity of liver inflammation and fibrosis (36–38). For instance, IL-6 induces the differentiation of naïve CD4^+^ T cells into Th17 cells, which in turn produces IL-17A that brings about hepatic IL-6 expression via MAPK signaling pathway (39), perpetuating IL-17A/Th17-mediated fibrosis. IL-6 knockout or neutralized mice fed with high fat diet exhibit reduced liver injury, which corroborates the deleterious role of IL-6 on NAFLD pathogenesis (40, 41). Interestingly, secretion of IL-18 and IL-10, cytokines reported to negatively regulate high fat diet-induced obesity and insulin resistance (42), and protect against hepatic steatosis (43), respectively, were increased in our HFHC-fed HIL mice. This increase could reflect a host immune response triggered to counteract the pro-inflammatory response induced by NAFLD or it may be a consequence of reduced sensitivity to these immune mediators (42, 44).

Insulin resistance is a primary risk factor associated with NAFLD in wildtype C57BL/6 mice (4). However, our HIL mice fed with HFHC diet remained insulin sensitive with increased secretion of total insulin. We attribute this discrepancy to the choice of HFHC diet used. Consistent with our data, Omar et al. reported that wildtype C57BL/6 mice placed on an 8-week Surwit high fat-high sucrose diet (D12331; Research Diet Inc) exhibited modest insulin resistance compared to severe insulin resistance in those on high fat diet (D12492; Research Diet Inc), and this difference was due to increased secretion of total insulin (22). Furthermore, NSG mice have been demonstrated to be more insulin sensitive when fed with high fat diet than C57BL/6 mice, while still developing hepatic steatosis (45, 46). Other surrogate biomarkers for NAFLD, including liver enzymes (ALT, AST, and GGT) and fibrotic markers (TGF-β1, TIMP-1), were not elevated in our NAFLD-HIL mouse model. While these biomarkers may be indicative of NAFLD, accurate diagnosis requires liver biopsy and histological assessment (47, 48). For instance, a retrospective study by Mofrad et al. revealed that all histological stages of NAFLD can be found in patients with normal ALT levels (47). The non-significant decrease in inflammation marker gene, *TNFA*, in total liver RNA of HFHC-fed mice remains enigmatic although a plausible explanation could be the reduced proportion of monocytes/macrophages (the major producers of TNFα) in the liver.

Finally, although our HIL mice were reconstituted with human CD34^+^ fetal liver cells (rich in hematopoietic stem/progenitor cells), the contribution of human hepatic cells to HFHC diet-induced pathologies was not defined due to low reconstitution (∼5–10%) (25). That said, we observed the up-regulation of human fibrotic markers *ACTA2* and *COL1A1*, suggesting that human hepatic stellate cells were present, activated and contributing to fibrosis. To better define the role of human hepatic cells in HFHC diet-induced pathologies, further improvement in the reconstitution of these cells in HIL mice is needed. Nonetheless, our current model could be useful for the pre-clinical evaluation of potential drugs targeting human immune cells.

In summary, we described a humanized mouse model for diet-induced NAFLD and showed that subsets of CD4^+^ T cells, specifically IL-17A-secreting Th17 and IFNγ -secreting Th1, play a key role in the progression of steatosis to fibrosis. To our knowledge, this is the first humanized mouse model for NAFLD, which could promote better understanding of NAFLD in humans, and potentially enable precise pre-clinical assessment of novel immunomodulatory treatments.

## Data Availability Statement

All datasets presented in this study are included in the article/Supplementary Material.

## Ethics Statement

The studies involving human participants were reviewed and approved by KK Women’s and Children’s Hospital (Singapore; CIRB Ref: 2013/837/D), National University Hospital (Singapore; NHG DSRB Ref: 2014/00231), and Gleneagles Hospital (Singapore; NUS-IRB Ref: 13-273E). Written informed consent to participate in this study was provided by the participants’ legal guardian/next of kin. The animal study was reviewed and approved by Institutional Animal Care and Use Committee, A^∗^STAR (IACUC number 181367).

## Author Contributions

ZH and QC designed research. ZH, JT, Y-SL, ST, XC, WT, ML, KY, FL, and ZZ performed research. ZH, JT, ST, XC, WT, EC, and QC analyzed data. YF, KC, LS, SC, C-LC, GL, YD, Y-SC, SL, JC, and KGC provided research tools and reagents, and contributed to the manuscript. ZH, JT, KGC, and QC wrote the manuscript. QC conceived the study and supervised the project. All authors contributed to the article and approved the submitted version.

## Conflict of Interest

C-LC is employed by Merck & Co., Inc., Kenilworth, NJ, United States. The remaining authors declare that the research was conducted in the absence of any commercial or financial relationships that could be construed as a potential conflict of interest.

## Abbreviations

ALT: alanine aminotransferase
AST: aspartate aminotransferase
CM: central memory
EM: effector memory
FF: fast food
FG/SR: Fast Green/Sirius Red
GGT: γ -glutamyl transferase
H&E: Hematoxylin and Eosin
IHC: immunohistochemistry
MCD: methionine choline-deficient
mDC: myeloid dendritic cell
NAFLD: non-alcoholic fatty liver disease
NASH: non-alcoholic steatohepatitis
NK: Natural killer
NSG: NOD-*scid Il2r γ ^*null*^*
pDC: plasmacytoid dendritic cell
RBC: red blood cell
ROS: reactive oxygen species
TEMRA: effector memory re-expressing CD45RA
Treg: regulatory T cell.
