# Supplementary material for: CD4+ T Cells Mediate the Development of Liver Fibrosis in High Fat Diet-Induced NAFLD in Humanized Mice
Source: Front Immunol. 2020 Sep 11;11:580968. doi: 10.3389/fimmu.2020.580968 (PMC7516019; doi:10.3389/fimmu.2020.580968)
Supplement: Supplementary file 1 [file Data_Sheet_1.PDF]

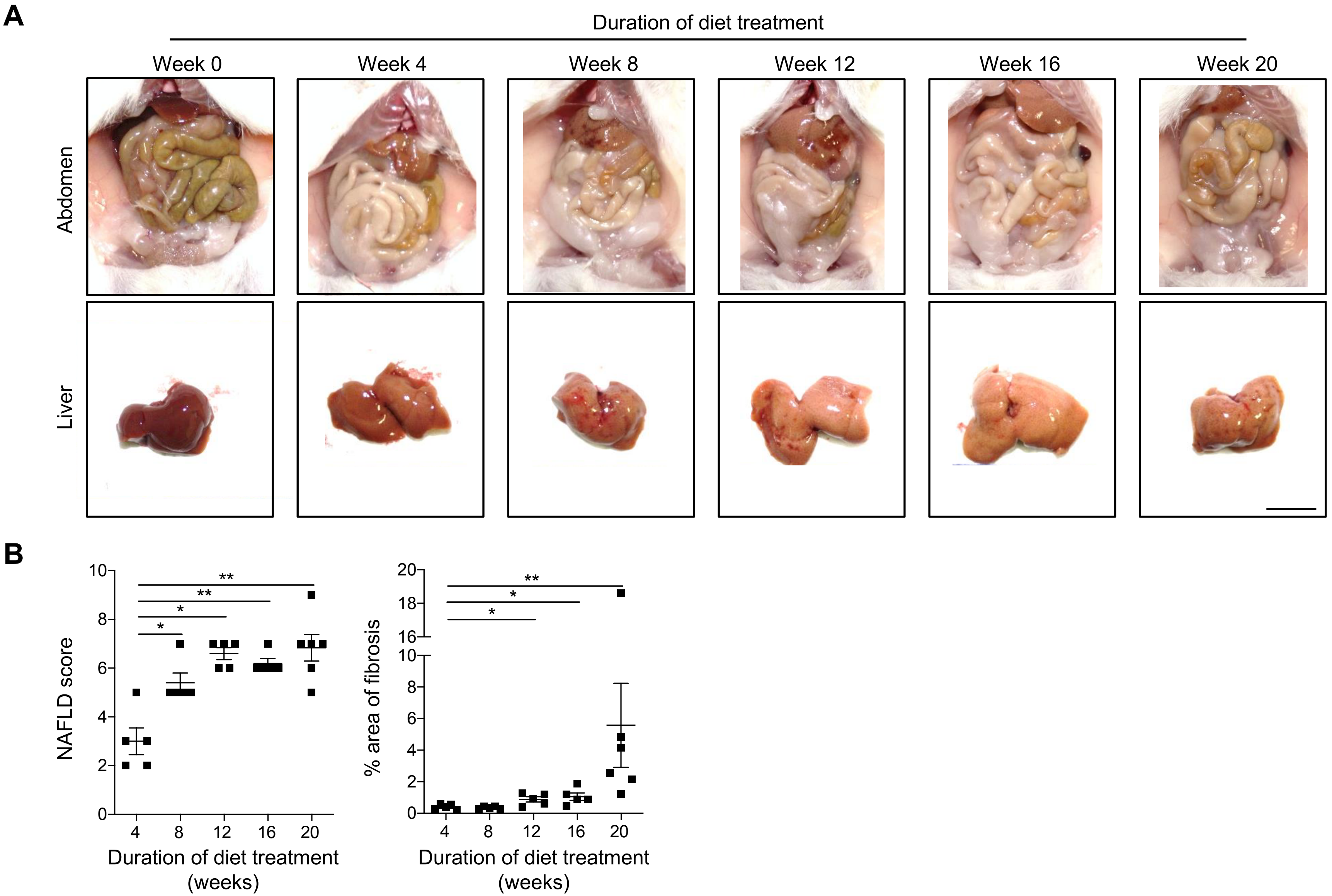

Figure S1. Increased abdominal fat and liver damage in HIL mice fed with HFHC diet over time. Ten to 12-week-old HIL mice were given *ad libitum* access to HFHC diet over a period of 20 weeks and sacrificed at weeks 0, 4, 8, 12, 16 and 20 of diet treatment. **(A)** Images are representative of 3-6 HIL mice at each time point. Scale bar: 1 cm. **(B)** Longitudinal NAFLD score and % area of liver fibrosis in HIL mice fed with HFHC diet at weeks 4 (n = 5), 8 (n = 5), 12 (n = 5), 16 (n = 5) and 20 (n = 6) of diet treatment. Data are presented as mean ± SEM. Pairwise comparison to week 4 of diet treatment group was performed using two-tailed Mann-Whitney U test; *; $p < 0.05$, **; $p < 0.01$.

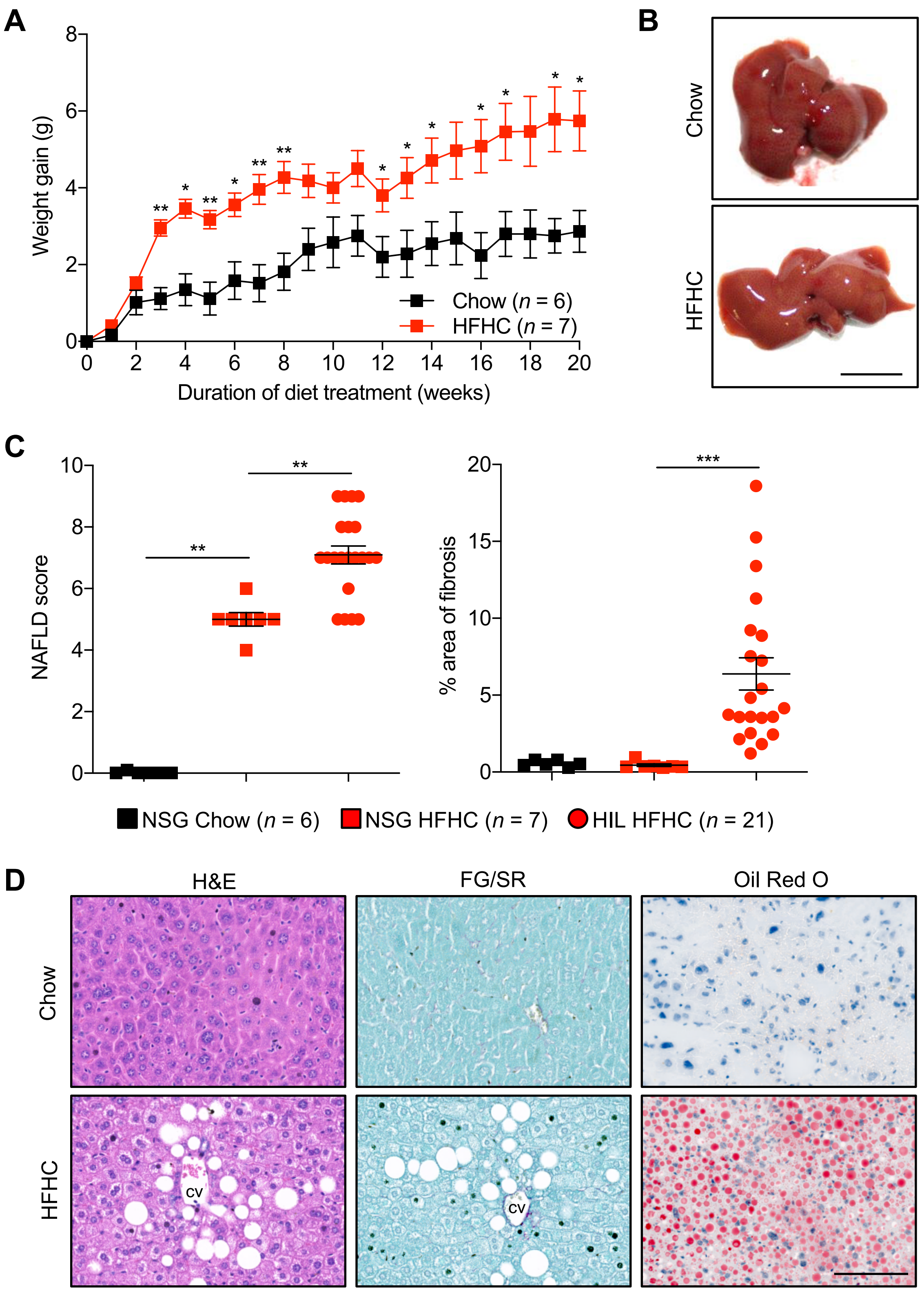

Figure S2. Liver damage is less severe in NSG mice fed with HFHC diet. Ten to 12-week-old NSG mice were given *ad libitum* access to HFHC diet over a period of 20 weeks. **(A)** Weight gain over a period of 20 weeks. Weight gain of NSG mice fed with chow diet (chow; black line; *n* = 6) and HFHC diet (HFHC; red line; *n* = 7) was normalized to weight at week 0. Data are presented as mean weight gain ± SEM. Two-tailed Mann-Whitney *U* test; *; $p < 0.05$, **; $p < 0.01$. **(B)** Liver from NSG mice fed with chow or HFHC diet. Images are representative of 6-7 NSG mice per diet group at week 20. Scale bar: 1 cm. **(C)** Comparison of NAFLD score and % area of liver fibrosis in NSG mice fed with chow (*n* = 6) or HFHC diet (*n* = 7), and HIL mice fed with HFHC diet (*n* = 21) at week 20. Data are presented as mean ± SEM. Pairwise comparison to NSG mice fed with HFHC diet was performed using two-tailed Mann-Whitney *U* test; **; $p < 0.01$, ***; $p < 0.001$. **(D)** Steatosis, but not inflammation and fibrosis, was observed in liver of NSG mice at week 20 of HFHC diet treatment. Images are representative of 6-7 NSG mice per diet group. Scale bar: 100 µm. cv, central vein.

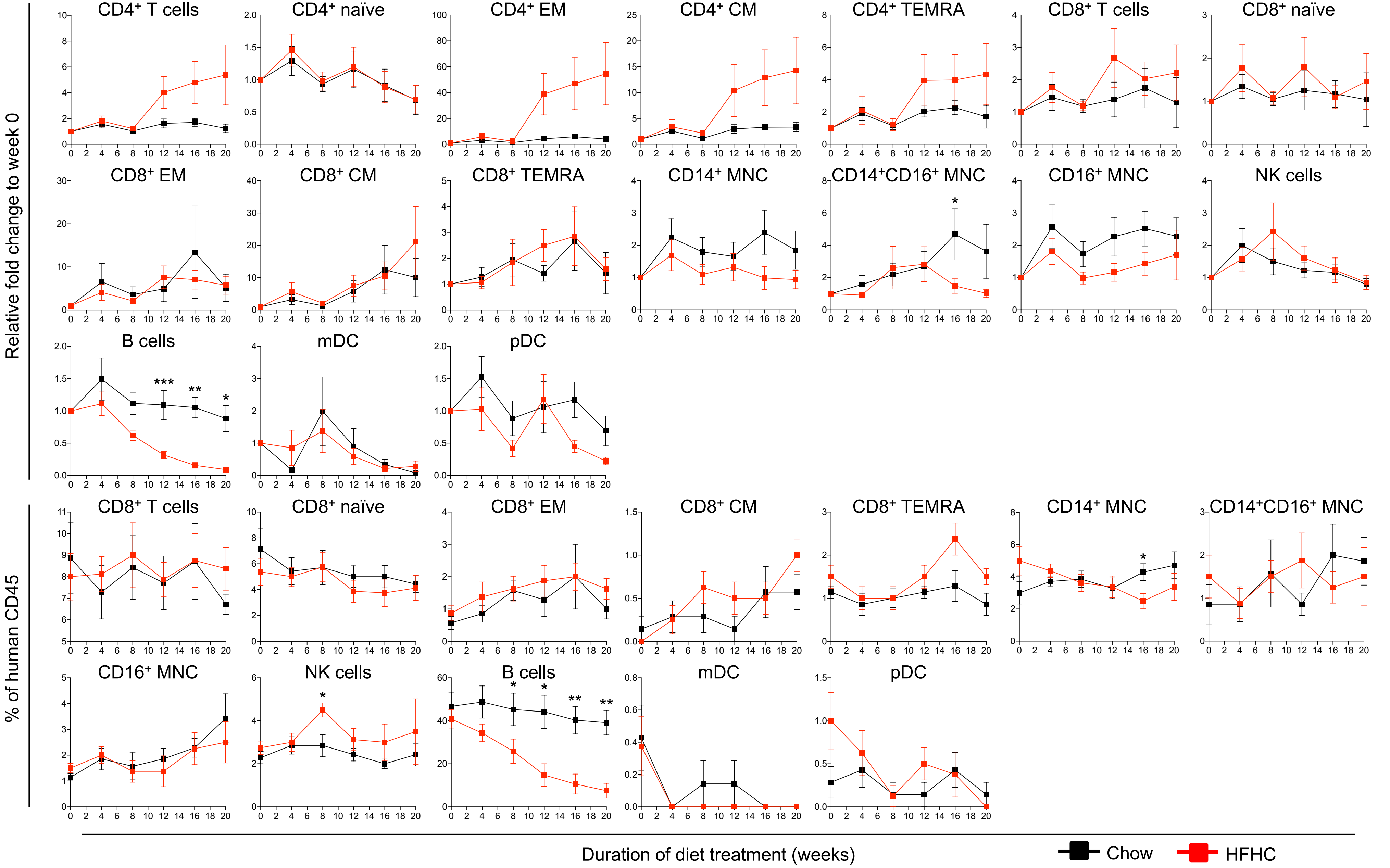

Figure S3. Longitudinal change in peripheral immune cell subsets in HIL mice fed with chow or HFHC diet. Ten to 12-week-old HIL mice were given *ad libitum* access to either chow diet (n = 7) or HFHC diet (n = 8) over a period of 20 weeks. Monocytes (MNC), Natural killer cells (NK), Dendritic cells (mDC and pDC). Data are presented as mean relative fold change ± SEM after normalizing the absolute count for each immune subset at each time point to week 0 (top panels) and mean % relative to human CD45 ± SEM at each time point (bottom panels). Two-tailed Mann-Whitney *U* test; *; $p < 0.05$, **; $p < 0.01$, ***; $p < 0.001$.

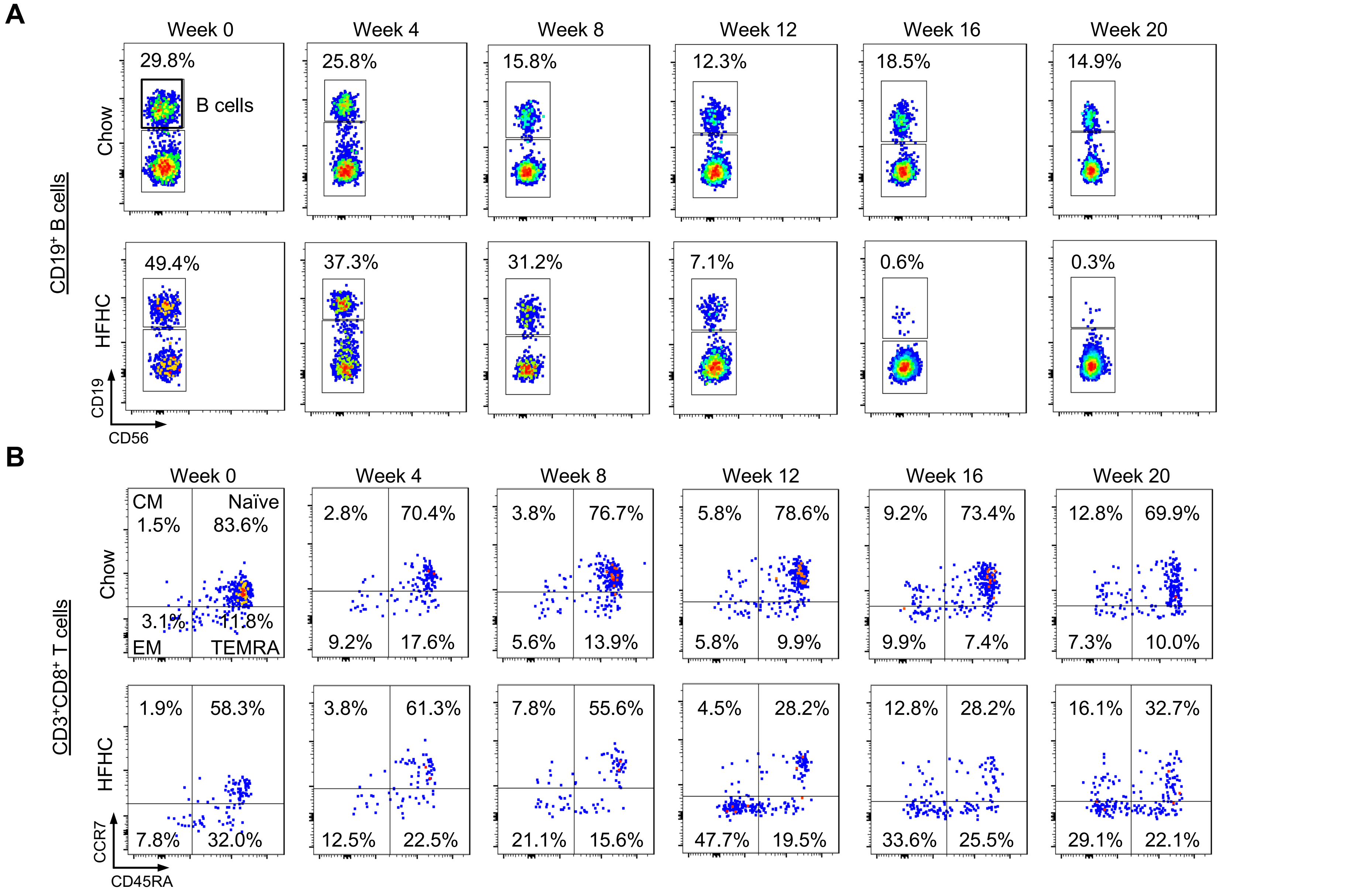

Figure S4. Peripheral B cells and CD8⁺ T cells in HIL mice fed with chow or HFHC diet. Blood was drawn from HIL mice fed with chow diet (*n* = 7) or HFHC diet (*n* = 8) at weeks 0, 4, 8, 12, 16 and 20, and immunophenotyped using flow cytometry. **(A)** Representative flow cytometry plots to illustrate the longitudinal change in $CD19^+$ B cells. The proportion of cells is presented as % relative to total human CD45 cells. **(B)** Representative flow cytometry plots to illustrate the longitudinal change in $CD8^+$ T cell naïve and memory subsets. The proportion of cells is presented as % relative to total $CD3^+CD8^+$ T cells. Human $CD3^+CD8^+$ T cells gated from human $CD45^+$ cells were further gated based on CCR7 and CD45RA expression for $CCR7^+CD45RA^+$ naïve, $CCR7^+CD45RA^-$ central memory (CM), $CCR7^-CD45RA^-$ effector memory (EM) and $CCR7^-CD45RA^+$ effector memory re-expressing CD45RA (TEMRA) cells.

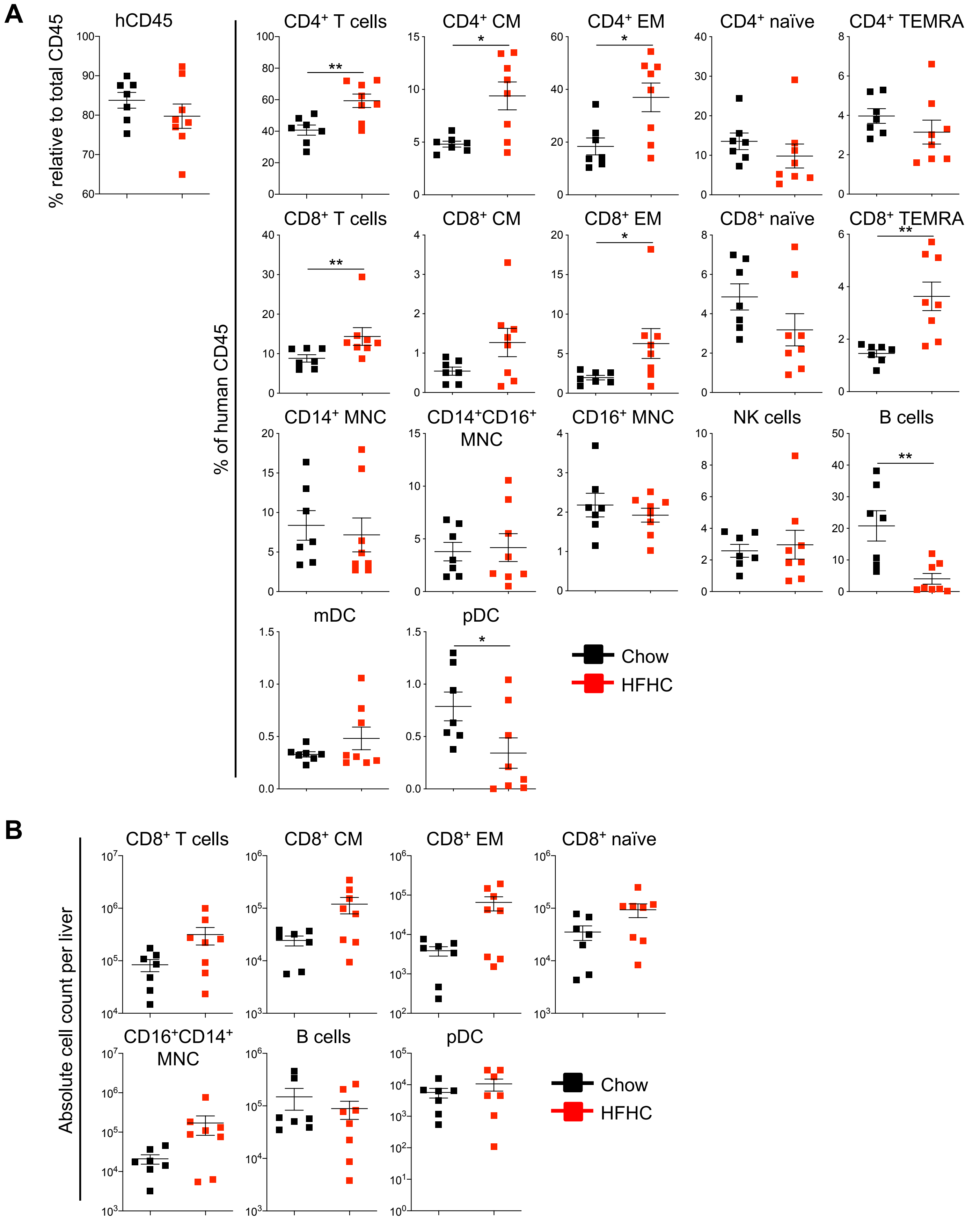

Figure S5. Profile of intrahepatic immune infiltrates in HIL mice fed with chow or HFHC diet. HIL mice given *ad libitum* access to either chow diet (n = 7) or HFHC diet (n = 8) were sacrificed and their liver harvested at week 20 of diet treatment. **(A)** Proportion of intrahepatic cellular infiltrates of each diet group at week 20. Proportion of human CD45 is presented as % relative to total CD45 cells, while the proportions of specific human immune cell subsets are presented as % relative to human CD45 cells. Data are presented as mean ± SEM. Two-tailed Mann-Whitney U test; *; $p < 0.05$, **; $p < 0.01$. **(B)** Absolute count of intrahepatic cellular infiltrates (statistically insignificant ones) of each diet group at week 20. Data are presented as mean absolute cell count per liver ± SEM. Two-tailed Mann-Whitney U test; $p > 0.05$.

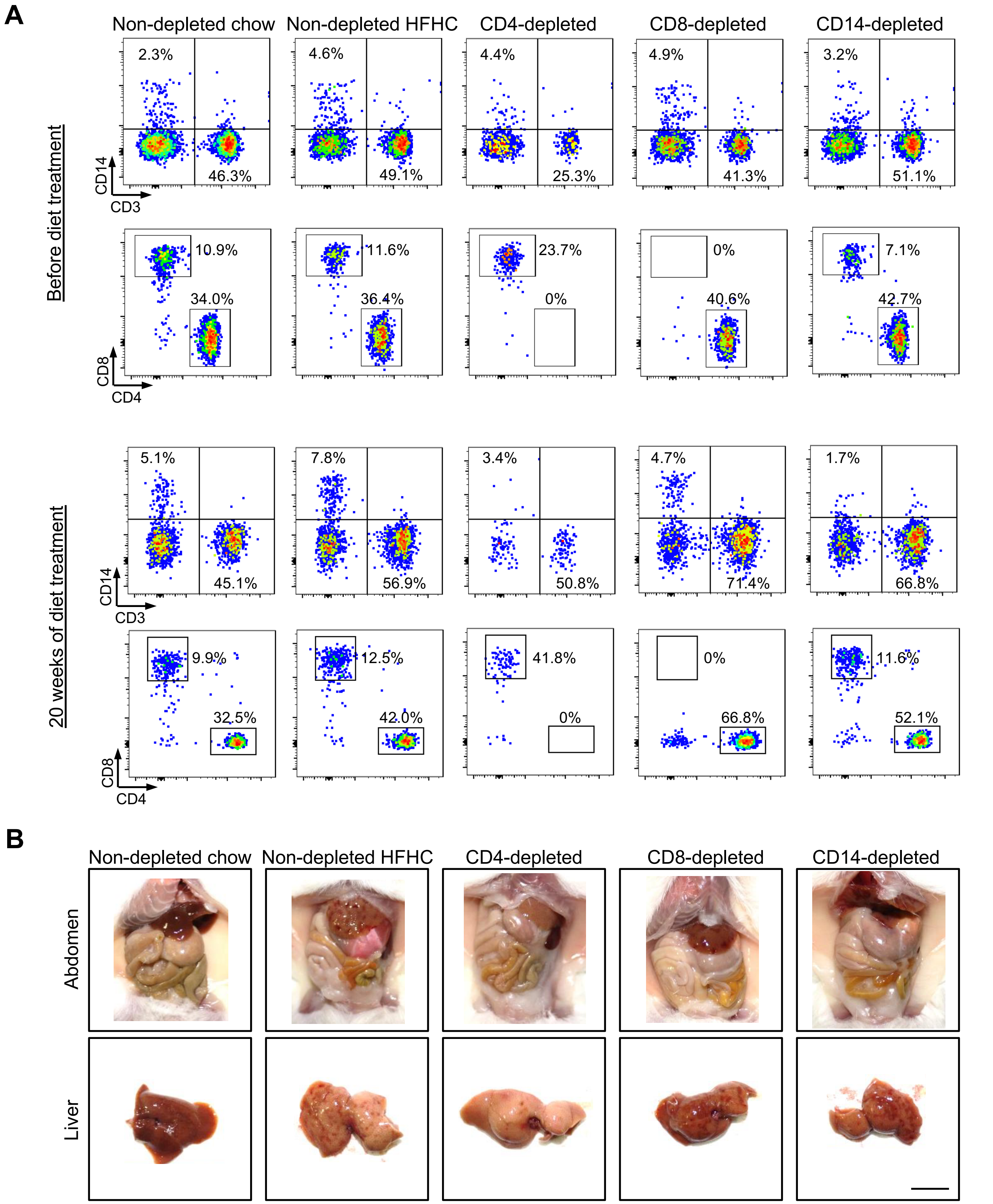

Figure S6. HIL mice depleted of CD4+ T cells and fed with HFHC diet had less damaged livers. HIL mice were intravenously injected with 50 µg of anti-human CD4, CD8 or CD14 depleting antibody one week before commencement of diet treatment. During the period of diet treatment, mice were intraperitoneally injected with 20 µg of respective depleting antibody weekly. **(A)** Peripheral blood profile of HIL mice depleted of either CD4, CD8, or CD14 cells before commencement of diet treatment (one week after first dose of depleting antibody injection) and at week 20 of diet treatment. Cell proportion is presented as % relative to human CD45 cells. Plots are representative of 4-9 HIL mice per group. **(B)** Effect of specific immune cells depletion on abdominal fat accumulation and liver pathology in HIL mice. Images are representative of 4-9 HIL mice per group. Scale bar: 1 cm.

Table S1. Human chimerism analysis in HIL mice at 10 weeks post-engraftment

	Peripheral blood chimerism (% hCD45)*		
FL†	Mean	Median	Range (min-max)
FL030216	60.4%	60.4%	39.3%-93.8%
FL0311YR	48.3%	48.5%	25.3%-72.3%
FL1108	31.9%	29.2%	16.5%-55.9%
FL1408	65.5%	65.5%	42.6%-89.6%
FL3012	39.2%	41.1%	12.5%-61.5%
FL2606	26.4%	27.4%	10.5%-43.1%
FL1911Q	40.6%	42.6%	26.3%-48.7%
FL220416Q	49.4%	55.2%	19.9%-66.0%
FL1707	47.8%	42.5%	27.8%-78.2%

*Refers to human CD45 relative to the sum of human and mouse CD45

†Fetal liver

Table S2. Distribution of histological NAFLD scores of mice at endpoint

		Histopathological features																	
		Macrovesicular steatosis*				Microvesicular steatosis*				Hypertrophy*				Inflammation†				Fibrosis‡	
Figure no.	Diet/Treatment group/Score	0	1	2	3	0	1	2	3	0	1	2	3	0	1	2	3	Absent	Present
Figure 1C, 6C and S2C	HIL chow (*n* =9)	9	0	0	0	9	0	0	0	9	0	0	0	8	1	0	0	9	0
	HIL HFHC (*n* = 21)	0	6	15	0	0	3	3	15	5	16	0	0	0	7	6	8	0	21
Figure 6C	CD4-depleted HFHC (*n* = 9)	0	1	8	0	0	1	2	6	5	4	0	0	9	0	0	0	9	0
	CD8-depleted HFHC (*n* = 5)	0	4	1	0	0	1	3	1	4	1	0	0	0	0	2	3	0	5
	CD14-depleted HFHC (*n* = 10)	0	5	5	0	0	2	1	7	4	6	0	0	0	4	2	4	0	10
Figure S1B	Week 0 (*n* = 3)	3	0	0	0	3	0	0	0	3	0	0	0	3	0	0	0	3	0
	Week 4 (*n* = 5)	0	4	1	0	0	4	1	0	2	3	0	0	0	5	0	0	5	0
	Week 8 (*n* = 5)	0	4	1	0	0	0	4	1	0	5	0	0	0	5	0	0	5	0
	Week 12 (*n* = 5)	0	2	3	0	0	0	0	5	0	5	0	0	0	5	0	0	5	0
	Week 16 (*n* = 5)	0	4	1	0	0	0	0	5	0	5	0	0	0	5	0	0	5	0
	Week 20 (*n* = 6)	0	2	4	0	0	0	2	4	1	5	0	0	0	3	2	1	0	6
Figure S2C	NSG chow (*n* = 6)	6	0	0	0	6	0	0	0	6	0	0	0	6	0	0	0	6	0
	NSG HFHC (*n* = 7)	0	0	7	0	0	0	1	6	6	1	0	0	7	0	0	0	7	0

*Steatosis, defined as macrovesicular steatosis and microvesicular steatosis, and hypertrophy were examined on H&E stained liver cross-sections at 5x magnification using the Zen 2 (blue edition; Zeiss) software and scored 0-3 based on the percentage of area affected: 0 (<5%), 1 (5-33%), 2 (33-66%), and 3 (>66%).

†Inflammation, defined as a cluster of more than five immune cells per focus, was examined on H&E stained liver cross-sections at 5x magnification using the Zen 2 (blue edition; Zeiss) software. Inflammation was evaluated at five different image fields and scored 0-3 based on the average number of immune foci per field: 0 (0.5 foci), 1 (0.5–1.0 foci), 2 (1.0–2.0 foci), and 3 (>2.0 foci).

‡Fibrosis was examined on Fast Green/Sirius Red stained liver cross-sections at 5x magnification using the Zen 2 (blue edition; Zeiss) software.

Table S3. Primer sequences used for qRT-PCR

Primer name	Forward primer sequence (5' → 3')	Reverse primer sequence (5' → 3')
TGFB1	CGC TAA GGC GAA AGC CCT CAA TTT	ACA ATT CCT GGC GAT ACC TCA GCA
ACTA2	AGG CAC CCC TGA ACC CCA A	CAG CAC CGC CTG GAT AGC C
COL1A1	GGC TTC CCT GGT CTT CCT GG	CCA GGG GGT CCA GCC AAT
TIMP1	GGA ATG CAC AGT GTT TCC CTG	GGA AGC CCT TTT CAG AGC CT
TNFA	CAC TGA AAG CAT GAT CCG GG	CTG GGG AAC TCT TCC CTC TGG
GAPDH	AGG GCT GCT TTT AAC TCT GG	CCC CAC TTG ATT TTG GAG GGA

Table S4. Overview of patient cohort for analysis

Patient ID	Disease	Age	Gender	ALT (IU/L)	AST (IU/L)	Cirrhosis	Tumor
1	NASH/HCC	64	M	28	53	Yes	Yes
2	NASH/HCC	59	F	114	84	Yes	Yes
3	NASH/HCC	67	F	24	35	Yes	Yes
4	NASH/HCC	61	M	23	50	Yes	Yes
5	NASH/HCC	67	M	34	41	Yes	Yes
6	NASH/HCC	68	F	71	101	Yes	Yes

Non-cirrhotic/tumor portion of patient liver or liver explant was used for the analysis. Healthy liver perfusate was obtained from healthy living donor during liver transplantation (donor information not available).
